# Editorial: Women in psychiatry 2023: aging psychiatry

**DOI:** 10.3389/fpsyt.2025.1556398

**Published:** 2025-02-10

**Authors:** Elizabeta B. Mukaetova-Ladinska, Stella-Maria Paddick

**Affiliations:** ^1^ Department of Psychology and Visual Sciences, University of Leicester, Leicester, United Kingdom; ^2^ The Evington Centre, Leicester General Hospital Site, Leicester, United Kingdom; ^3^ Translational and Clinical Medicine, Newcastle University, Newcastle Upon Tyne, United Kingdom; ^4^ Department of Old Age Psychiatry, Gateshead Health National Health Service (NHS) Foundation Trust, Gateshead, United Kingdom

**Keywords:** women, ageing, mental health, wellbeing, academic psychiatry

Historically, women have faced many obstacles when entering professional life, irrespective of their chosen disciplines. Henry Maudsley, in his ‘Sex in Mind and in Education’, published in 1871, expressed the societal view on female progression to equality. For him, then a President of the Medical Psychological Association, a forerunner of the UK Royal College of Psychiatry, education of women was detrimental to their health, having a negative impact on their reproductive functions, and resulting in infertility. Fifty years later, pioneering psychological scientist Lillian Moller Gilbreth proved him wrong. She balanced family life and raising 12 children with a successful career as an industrial psychologist, inventor, college professor, filmmaker, and author of her own and co-authored books.

Her example was followed by a string of women who have left a lasting legacy on modern mental health and wellbeing: Anna Freud, a pioneer in children and young people’s mental health, Elisabeth Kübler-Ross^1^, a pioneer in near-death studies^2^ and the “Kübler-Ross” model^3^ of grieving, Kay Redfield Jamison, a leading authority on bipolar disorder, Lorna Wing who coined the term of autism spectrum and revolutionized thinking about autism, Nancy Andreas and her pioneering implementation of magnetic resonance imaging in mental illness (schizophrenia), just to name few. Basic neurobiological research attracted several successful female scientists. Neuroscientists, like Rita Levi-Montalcini, a Nobel Prize laureate, Virginia Lee and Elaine Perry and neuropathologist Margaret Esiri, contributed to identifying and characterizing proteins that play a role in major psychiatric diseases, such as depression and schizophrenia (neuritic growth factor), pathological inclusions (tau protein neurofibrillary tangles and alpha synuclein inclusions, Lewy Bodies) in neurodegenerative disorders (i.e. Alzheimer’s disease, Parkinson’s disease and Dementia with Lewy Bodies), and the development of anti-dementia drugs. These advances have been largely underpinned by Carol Brayne’s dementia epidemiological studies.

Having these beacons of excellence sadly has not yet resulted in narrowing the gender gap in academia and academic productivity. Women remain still under-represented in senior leadership roles, in both clinical and academic settings. This comes as a surprise since women tend to value work-life balance, opportunities for comprehensive and holistic caregiving, and the ability to address the psychological and social aspects of medicine^4^. It is, thus not surprising that psychiatry and psychology have a strong female representation. The most recent 2024 survey of 7000 USA physicians (58% women)^5^ confirm this, finding that psychiatrist value their relationship with patients and helping them.

Despite the increasing percentage of women in academic psychiatry posts (more than doubled) within the last 20 years in the UK), only 20% of senior academic posts at a professorial level were occupied by women (1). As publication activity reflects both leadership and participation in academia, the review by Hart et al. (2) offers an interesting analysis. Thus, whereas half of published authors had a female first author, slightly over one third had a senior (last) female author, with women having slower rates of transition to the last author position and requiring 20-25 years to achieve parity in senior authorship. Based on the contribution in the Oxford Textbook of Old Age Psychiatry (2020), out of 122 contributors, 46 (37.7%) were women, with only 13 out of 58 book chapter written only by women (3). Interestingly, articles with women as last authors were significantly more likely than those with men as last authors to have a woman as first author (2).

The relative absence of women in these senior scientific and leadership roles arguably affects equity in knowledge and outcomes of psychiatric disorders. There are well recognized diagnostic gender biases particularly in the case of personality disorders (4) and recognition of neurodevelopmental disorders and neurodivergence (5). Similarly, there has been a historical lack of representation of female subjects in psychopharmacological studies and pharmacological trials, due to potential complexity of data analysis due to effects of the menstrual cycle (6). Though this is now improving, this has limited our knowledge of sex-specific psychotherapeutic effects and potential for adverse drug reactions. In ageing psychiatry, there are signs of increasing senior female representation. Dementia is a condition which disproportionately affects women, both as individuals affected, and caregivers. Currently, two of the senior scientific leaders in dementia risk reduction worldwide are women, including Gill Livingston, Chair of the Lancet Commission on dementia risk reduction, and Mia Kivipelto, lead of the FINGERS dementia risk reduction programmes.

What is the focus of current ageing psychiatry research by women? The articles in this Research Topic “Women in Psychiatry’ extend beyond a focus on caregiving. Female authors co-authored the papers as first author (n=4) (Pham-Scottez et al., Pfister et al., Kuck et al. and Armstrong et al.) and/or senior author (n=2) (Kuck et al. and Leon et al.), with one paper having both female authors (Kuck et al.). The articles not only address the mental health of older people but provide further insight about prevention of age-associated cognitive functioning, and neuropsychological coping with major life changing events, i.e. pandemic and loss of reproductive role in life with ageing, turning their attention to practical interventions to enhance wellbeing. These apparently diverse studies have a unifying theme, which is that of resilience. Furthermore, they have, directly and/or indirectly identified ways of enhancing resilience, via combating loneliness, age-associated infection and endocrinological changes, whilst enhancing executive function. Via analyzing the current situations in geriatric medicine, psychiatry and psychology, they have underlined the need to promote alternatives and opportunities for all, with the solutions being included in public and social health interventions and policies.

How relevant is this to women in old age psychiatry in 2023? The above studies have highlighted that promoting healthy lifestyles positively benefits cognition. This helps individuals in dealing with adversity and withstanding stress, qualities required in any successful leadership. As such, resilience can help bridge the gender gap that exists globally, including academia. Thus the latest 2024 global gender gap score for all 146 countries included in the latest 2024 edition stands at 68.5%^6^, meaning that over the last 18 years, the gap has improved by 0.1 points. With this rate, it will take another 134 years to reach full parity (or roughly five generations beyond the 2030 Sustainable Development Goal target). We must act now – as per this report, it is not female education and professional inability that contribute to the latter. We must embrace what decades of research have demonstrated - women leaders help increase productivity, enhance collaboration, inspire organizational dedication, and improve fairness. These values have a positive impact on the academic productivity, achievements, retention of academic staff and overall improvement of professional output. The growing number of older people worldwide deserves their expertise.

## Footnotes

1
https://www.ranker.com/review/elisabeth-kübler-ross/927782.
